# Myosin light chain of shark fast skeletal muscle exhibits intrinsic urea-resistibility

**DOI:** 10.1038/s41598-023-32228-w

**Published:** 2023-03-25

**Authors:** Satoshi Kanoh, Takayuki Noma, Hirotaka Ito, Masatomo Tsureyama, Daisuke Funabara

**Affiliations:** 1 Graduate School of Bioresources, Mie University, Tsu, Mie 514-8507 Japan; 2 Present Address: Kogakkan High School, Ise, Mie 516−8577 Japan; 3 Present Address: ASGEN Pharmaceutical Co., Ltd., Mizunami, Gifu 509-6104 Japan; 4 Present Address: Kracie Foods, Ltd., Minato, Tokyo, 108-8080 Japan

**Keywords:** Proteins, Contractile proteins

## Abstract

Marine elasmobranch fish contain urea, a protein denaturant, in their bodies. The urea-trimethylamine *N*-oxide (TMAO) counteraction mechanism contributes to urea-resistibility, where TMAO compensates for protein denaturation by urea. However, previous studies revealed that shark fast skeletal muscle myosin exhibits native activity at physiological urea concentrations in the absence of TMAO, suggesting that shark myosin has urea-resistibility. In this study, we compared the urea-resistibility of myosin alkali light chains (A1-LC and A2-LC) from banded houndshark and carp by examining the α-helical content at various urea concentrations. The α-helical content of carp myosin A1-LC and A2-LC gradually decreased as urea concentrations increased to 2 M. In contrast, the α-helical content of banded houndshark A1-LC increased between 0 and 0.5 M urea, and the α-helical content of A2-LC remained constant until 0.5 M urea. We determined the full-length sequences of the banded houndshark myosin light chains (A1-LC, A2-LC and DTNB-LC). Hydrophilicity analysis revealed that the N-terminal region (residues 28–34) of A1-LC from banded houndshark is more hydrophilic than the corresponding region of A1-LC from carp. These findings support the notion that shark myosin exhibits urea-resistibility independent of the urea-TMAO counteraction mechanism.

## Introduction

Marine elasmobranch fish have urea concentrations of 200–460 mM in their body for osmoregulation^1^. Banded houndshark *Triakis scyllium* contains 194 mM urea in the dorsal muscle, 195 mM in blood serum and 175 mM in the liver ^2^. Urea is a well-known protein denaturant, interfering with hydrogen bonds^3^, hydrophobic interactions^4^ and the structure of water^5^. Recent studies have employed molecular simulations to explore the atomic mechanisms by which urea destabilizes proteins. Some studies has been reported that urea interacts directly with polar residues and the peptide backbone, stabilizing nonnative conformations^6^, preferential hydrogen bond formation between the urea carbonyl and the backbone amides that contributes to the breaking of intrabackbone hydrogen bonds^7^, and urea destabilizes proteins by forming hydrogen bonds to the peptide group^8^

Marine elasmobranch fish swim like teleosts with small amounts of urea in their body, indicating that proteins within their body can tolerate urea. The lens protein of dogfish *Mustelus canis* does not precipitate under 10 °C in the presence of urea but does in the absence of urea^9^. The oxygen affinity of clearnose skate *Raja eglanteria* hemoglobin is maintained up to 5 M urea^10^. Marine elasmobranch lactate dehydrogenases require physiological urea concentrations to be active at the same level as their teleost counterparts^11^.

Currently, the urea-resistibility of elasmobranch fish can be explained by two strategies. The first strategy involves retaining an unusual amount of methylamine, such as betaine and trimethylamine *N*-oxide (TMAO), that marine elasmobranch fish use as osmolytes in the body to neutralize the effects of urea (urea-methylamine counteraction)^12^. The simulation method showed that TMAO makes few direct interactions with the protein. Instead, it prevents unfolding of the protein by structuring the solvent^13^, and TMAO can counteract the denaturing effects of urea by inhibiting protein–urea preferential interaction^14^. The other strategy is the evolution of a protein structure that resists urea denaturation. Banded houndshark fast skeletal myofibrils show urea-resistibility for Ca^2+^- and Mg^2+^-ATPase activities, suggesting that myofibril components, especially myosin molecules, have native urea-resistibility^15,16^. Banded houndshark myosin has been shown to exhibit native ATPase activity in the presence of 0.3 M urea, which is close to the physiological concentration in muscle tissue, whereas carp myosin loses 70% of its ATPase activity at this urea concentration^16^. For banded houndshark myosin, TMAO cannot neutralize the urea effect on myosin ATPase activity but actually inhibits the activity of this enzyme^15^. These findings might indicate that the molecular structure of elasmobranch myosin provides urea-resistibility to myosin molecules.

Myosin is a hexamer of two identical heavy chains, with the head regions providing the ATPase activity and two sets of light chains. The light chains are categorized into alkali or 5,5'-dithiobis(2-nitrobenzoic acid) (DTNB) because they detach from myosin heavy chains following treatment with alkali or DTNB solutions, respectively. Two types of alkali light chains exist, namely A1 and A2 light chains (A1-LC and A2-LC). The primary structures of A1-LC and A2-LC have high sequence identities, except that A1-LC has an N-terminal extension of about 40 amino acids rich in hydrophobic residues. In mammals, A1-LC and A2-LC originate from the same gene^17–19^, whereas in teleosts, the A1-LC and A2-LC genes exist on different loci^20–22^. The nomenclature of myosin light chain is a little confusing: the ‘alkali’ light chain is also called the ‘essential’ light chain, and the ‘DTNB’ light chain is also called the ‘regulatory’ light chain^23^.

Chymotryptic digestion of myosin heavy chain separates it into subfragment-1 (S1), which is responsible for ATPase activity, and the rod, which participates in forming myosin filaments. Surface hydrophobicity changes to banded houndshark S1 and rod induced upon exposure to urea suggest that the region connecting S1 and rod contributes to the urea-resistibility of myosin^24^. The connecting part is where myosin light chains intertwine, suggesting that myosin light chains may play a role in urea-resistibility by binding this region. A reduction in the velocity of actin filaments without decreasing ATPase activity significantly is observed when myosin heavy chains are used without light chains^25^. The re-addition of the light chains to the myosin molecule restores the actin sliding velocity^25^. These findings indicate that, in elasmobranch fish, myosin light chains provide steric stability to myosin molecules, thus allowing them to function as they work in teleosts even under high urea concentrations. Unfortunately, few studies are available that define the function of the myosin light chains in the urea-resistibility of elasmobranch fish.

In the present study, we investigated the urea-resistibility of banded houndshark A1-LC and A2-LC by circular dichroism (CD) analysis using α-helical content of the light chains as an indicator. We determined the sequences of banded houndshark A1-, A2- and DTNB-LCs to unveil their molecular characteristics. We discuss the role of myosin light chains in myosin urea-resistibility.

## Results

### Preparation of myosin light chains

Using DEAE-Toyopearl and phenyl-5PW chromatography, we successfully isolated myosin A1- and A2-LCs from banded houndshark and carp fast skeletal muscle (Fig. S1). The purity of these LCs was sufficient for CD analysis to investigate the urea-resistibility using their α-helical content as an indicator. Urea-resistibility of shark myosin light chain was investigated compared to that of carp purified under the same conditions.

### CD analysis of myosin A1 and A2 light chains in the presence of urea

**Figure 1:**
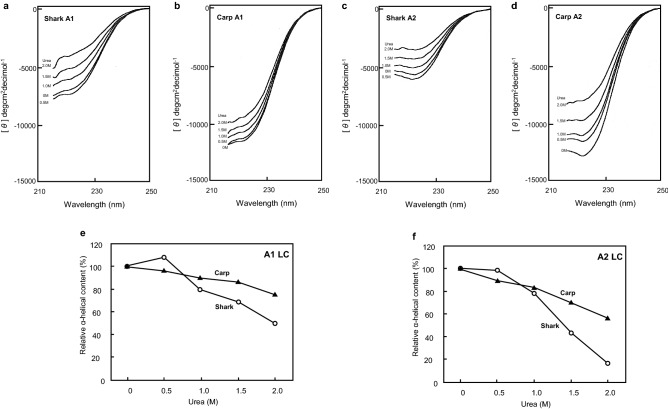
CD analysis of myosin A1 and A2 light chains from banded houndshark and carp in the presence of urea. (**a**–**d**) CD spectra of the A1 and A2 light chains (A1-LC, A2-LC) of shark and carp. The representative data were shown. (**e**, **f**) changes in the relative α-helical content of shark and carp myosin A1- and A2-LCs in the presence of urea. Open circles and filled triangles represent the data from shark and carp, respectively.

We confirmed that the purified A1-LCs from banded houndshark and carp were suitable for the CD analysis by measuring in the absence of urea (Fig. S2). CD measurement of A1-LC and A2-LC was carried out over the range of 205–250 nm. Note, however, that relative intense spectral noise was detected under 215 nm because of the strong absorbance by urea. Thus, CD spectra above 215 nm are shown (Fig. 1a–d). The α-helical content at each urea concentration relative to the α-helical content in the absence of urea was calculated (Fig. S3, Fig. 1e, f). The α-helical content of carp A1-LC gradually decreased as the urea concentration increased, whereas the α-helical content of banded houndshark A1-LC increased slightly at 0.5 M urea and then progressively decreased as the concentration of urea increased above 0.5 M (Fig. 1e). The α-helical content of carp A2-LC gradually decreased as the urea concentration increased, whereas the α-helical content of shark A2-LC was retained to 0.5 M urea and then progressively decreased as the urea concentration increased (Fig. 1f).

### Internal amino acid sequencing of banded houndshark myosin A1 and A2 light chains

Sequencing of banded houndshark A1-LC digested with TPCK-treated trypsin gave two sequences: AAAAPAPAAAPPPPEPPKPKEPSVDLSKVKIEFSAEQQEDF and ILNNPSTEDMTSKAIEFDQFLPMLQTMANNKEQGS (Fig. S4). The sequence EDMTSKAIEFDQFLPMLQTMANNKEQ was obtained for A2-LC (Fig. S5).

### Molecular characteristic of banded houndshark myosin light chains

#### Myosin A1 light chain

**Figure 2:**
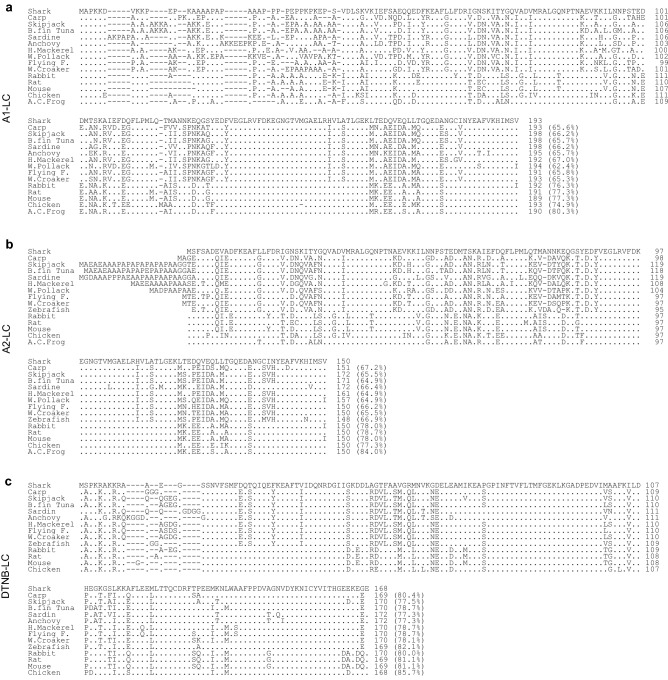
Multiple sequence alignment of the banded houndshark *Triakis scyllium* myosin light chains amino acid sequences determined in this study to those from other species. (**a**) A1 light chain, (**b**) A2 light chain, (**c**) DTNB light chain. Sequences for comparison were obtained from the DNA Data Bank of Japan: carp *Cyprinus carpio*, skipjack tuna *Katsuwonus pelamis*, bluefin tuna *Thunnus orientalis*, sardine *Sardinops melanostictus*, anchovy *Engraulis japonica*, horse mackerel *Trachurus japonicus*, walleye pollack *Gadus chalcogramma*, flying fish *Cypselurus agoo*, white croaker *Pennahia argentata*, zebrafish *Danio rerio,* rabbit *Oryctolagus cuniculus*, rat *Rattus norvegicus*, mouse *Mus musculus*, chicken *Gallus gallus* and African clawed frog *Xenopus laevis*. The numbers on the right side represent the amino acid sequence from the N-terminus. Gaps were inserted to optimize the sequence alignment. Dots indicate identical amino acid residues to those of shark myosin light chain. Percent sequence identities between shark myosin light chain and the other sequences are given in parentheses.

Immuno-screening of the banded houndshark fast skeletal muscle cDNA library using the anti-banded houndshark A1-LC antiserum yielded a cDNA clone encoding the full-length banded houndshark A1-LC nucleotide sequence of 1070 nucleotides (nt), which contains an open reading frame (ORF) of 579 nucleotides, a 5'-untranslated region of 53 nt and a 3'-untranslated region of 438 nt (Fig. S4). The ORF encodes a protein of 193 amino acid residues (aa) in length with a predicted molecular mass of 21.2 kDa and a calculated isoelectric point (pI) of 4.86. The protein sequence contained the sequences determined by N-terminal protein sequencing, indicating that the protein purified was A1-LC. Comparison of the amino acid sequence of banded houndshark A1-LC with sequences from other species gave the following sequence identities: 65.6% (carp), 66.2% (skipjack), 65.7% (bluefin tuna), 66.2% (sardine), 65.7% (anchovy), 67.0% (horse mackerel), 62.4% (walleye pollack), 65.8% (flying fish) and 65.3% (white croaker) in teleosts, and 76.3% (rabbit), 77.3% (rat), 77.3% (mouse), 74.9% (chicken) and 80.3% (African clawed frog) in other vertebrates (Fig. 2a).

#### Myosin A2 light chain

**Figure 3:**
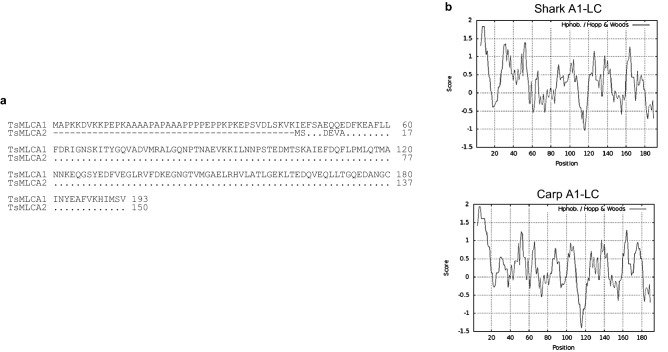
Hydrophicility analysis of the A1 and A2-LCs of banded houndshark *Triakis scyllium*. (**a**) comparison of the amino acid sequences of myosin A1- and A2-light chains. The numbers on the right side represent the amino acid sequence from the N-terminus. Gaps were inserted to optimize the sequence alignment. Dots indicate identical amino acid residues between them. (**b**) hydrophilicity values of the shark (upper) and carp (lower) myosin A1 light chains.

Immuno-screening of the banded houndshark fast skeletal muscle cDNA library using the anti-banded houndshark A2-LC antiserum yielded a cDNA clone encoding the full-length banded houndshark A2-LC nucleotide sequence of 919 nt, which contains an ORF of 450 nt, a 5'-untranslated region of 26 nt and a 3'-untranslated region of 443 nt (Fig. S5). The ORF encodes a protein of 150 aa in length with a predicted molecular mass of 16.7 kDa and a calculated pI of 4.46. The protein sequence contained the sequence determined by N-terminal protein sequencing, indicating that the protein purified was A2-LC. Comparison of the amino acid sequence of banded houndshark A2-LC with sequences from other species gave the following sequence identities: 67.2% (carp), 65.5% (skipjack), 64.9% (bluefin tuna), 66.4% (sardine), 64.9% (horse mackerel), 64.9% (walleye pollack), 66.2% (flying fish), 65.5% (white croaker) and 66.9% (zebrafish) in teleosts, and 78.0% (rabbit), 78.7% (rat), 78.0% (mouse), 77.3% (chicken) and 84.0% (African clawed frog) in other vertebrates (Fig. 2b). Shark A2-LC is 43 residues shorter than shark A1-LC (Fig. 3a). The extended N-terminal region of A1 is rich in alanine and proline. We found the nucleotide sequences encoded near identical amino acid sequences for the ORFs of A1-LC and A2-LC, and 3'-untranslated regions are identical between the *TsMLC-A1* and *TsMLC-A2* genes, suggesting these genes are produced from a single gene, as observed for mammals.

#### Myosin DTNB light chain

Immuno-screening of the banded houndshark fast skeletal muscle cDNA library using the anti-banded houndshark myosin antiserum yielded a cDNA clone encoding the full-length banded houndshark DTNB-LC nucleotide sequence of > 1400 nt, with the 3'-end region including a poly-A tail that was not sequenced because of a sequence rich in G (Fig. S6). The nucleotide sequence contains an ORF of 504 nt, which encodes a protein of 168 aa in length with a predicted molecular mass of 18.7 kDa and a calculated pI of 4.92. A BLAST search with the sequence identified this protein as a myosin DTNB light chain. Comparison of the banded houndshark myosin DTNB light chain sequence with sequences from other species showed the following sequence identities: 80.4% (carp), 77.5% (skipjack), 78.7% (bluefin tuna), 77.3% (sardine), 77.3% (anchovy), 78.7% (horse mackerel), 78.7% (flying fish), 78.1% (white croaker) and 82.1% (zebrafish) in teleosts, and 80.0% (rabbit ), 81.1% (rat), 81.1% (mouse) and 85.7% (chicken) in other vertebrates (Fig. 2c).

#### Hydrophilicity of banded houndshark myosin light chain

Since urea affects the hydrophilic and hydrophobic groups of proteins, we calculated the hydrophilicity value of the banded houndshark A1-LC and compared it with that of carp A1-LC (Fig. 3b). Hydrophilic patterns revealed that the N-terminal region (residues 28–34) is highly hydrophilic in banded houndshark A1-LC, which contrasts with the lower hydrophilicity observed for the same region in carp A1-LC. This region may be responsible for the urea-resistibility of banded houndshark myosin. The banded houndshark and carp A2-LCs show almost identical hydrophilicity patterns of each of A1-LC lacking the N-terminal extension (Fig. S7).

## Discussion

CD analysis showed that the absolute α-helical content of banded houndshark myosin light chains was relatively lower than those of carp (Fig. S3), implying that the structure of shark ones is more unstable than that of carp ones. This result is consistent with the fact that shark myosin has a higher denaturation rate constant for heat than carp, and it occurs regardless of urea concentration^26^. For this reason, in this study, we used the relative α-helical content to compare the urea-resistibility of shark and carp myosin light chains.

CD analysis revealed that the α-helical structure of banded houndshark and carp A1-LCs changed as the urea concentration increased (Fig. 1e), and changes were also observed for banded houndshark and carp A2-LCs (Fig. 1f). The α-helical content at each urea concentration relative to the α-helical content in the absence of urea was calculated for the LCs. The relative α-helical content of carp A1- and A2-LCs gradually decreased as the urea concentration increased, indicating that urea caused denaturation of the LC structures. The banded houndshark A1-LC had the highest α-helical content in the presence of 0.5 M urea, which is near the physiological urea concentration of the shark. The α-helical content of banded houndshark A2-LC showed essentially no change up to 0.5 M urea. The α-helix content of banded houndshark lowerered than that of carp above 1.0 M urea. These findings are consistent with our previous study^15^, which demonstrated that banded houndshark myofibrils exhibit urea-resistibility and retain their ATPase activity up to a concentration of 0.6 M urea, while the ATPase activity of carp myofibrils rapidly declines upon the addition of urea. Furthermore, our results suggest that banded houndshark LCs are capable of exhibiting their native activity at physiological urea concentrations independent of TMAO. We hypothesize that shark A1- and A2-LCs acquire resistibility toward urea-induced denaturation because of their molecular structure based on their primary structure.

We determined the full-length sequences of banded houndshark A1-, A2- and DTNB-LCs (Figs. S4-S6). The banded houndshark A1-LC is 43 residues longer when compared with the sequence of banded houndshark A2-LC, as observed for A1-LCs from various species (Fig. 2a). The banded houndshark A1-LC shares a nearly identical sequence with A2-LC, with minor differences located at the N-terminus (Fig. 3a). It is challenging to reveal the relationship between urea resistibility and the primary structure of the myosin light chain from only the data obtained in this study. However, we will show one possibility to explain how the shark myosin light chain has got urea resistibility by the difference in the hydrophilicity between the shark and carp light chains (Fig. 3b). As shown in Fig. 1, the α-helical content of banded houndshark A1-LC reached a peak in the presence of 0.5 M urea, whereas the α-helical content of banded houndshark A2-LC did not increase at this urea concentration. This different behavior in urea-resistibility between A1- and A2-LCs may arise from the highly hydrophilic N-terminal sequence of A1-LC. Although carp A1 and A2-LCs exhibited lower urea-resistibility compared with the counterparts of banded houndshark, the carp A1-LC was more urea-resistant than the carp A2-LC, like the banded houndshark. The N-terminal region of A1-LC is well-known to stabilize the molecule structure compared with the A2-LCs, supporting our data that the shark and carp A1-LCs exhibit a more urea tolerance than the A2-LCs (Fig. 1e, f). The difference in urea-resistibility between the A1-LCs of banded shark and carp might be caused by the region (residues 28–34) of the N-terminal region of the banded houndshark A1-LC that is higher hydrophilicity than the corresponding region of the carp one. There are also differences in the hydrophilicity of residues 60–70 and 170–180 of A1-LC. These regions might contribute to the urea resistibility. Nevertheless, it is unaccountable how these work for now. To reveal it, we will determine which region contributes to shark urea resistibility using protein synthesized by the amino acid substitution or deletion system.

The urea-TMAO counteraction theory is widely accepted to explain how marine elasmobranch fish adapt to high urea concentrations in their bodies, which may denature proteins. However, the mechanism of how TMAO offsets urea-induced denaturation remains controversial. A study showed that TMAO may inhibit the preferential interaction of urea with proteins^14^. In contrast, there are studies showing that the urea-TMAO counteraction mechanism is not active for several proteins: dogfish shark *Squalus acanthias* hemoglobin^27^, aldose reductase^28^, amphibian 6-phosphofructo-l-kinase and pyruvate kinase^29^ and catalase^30^. For catalase, the urea-TMAO counteraction stabilized the protein structure but did not affect enzymatic activity.

Our previous studies revealed that protein molecules in sharks may exhibit intrinsic urea-resistibility rather than rely on counteractants such as TMAO. Urea did not affect banded houndshark myofibrillar Mg^2+^-ATPase activity, whereas TMAO suppressed the activity of this protein. At a 2:1 molar ratio of urea to TMAO, the Mg^2+^-ATPase activity was remarkably lower when compared with the activity of the protein in the presence of only urea^15^. Compared to carp myosin, surface hydrophobicity of the banded houndshark myosin molecule was shown to gradually decrease as the urea concentration increased and eventually reached 62% at 2 M urea, indicating that banded houndshark myosin is tolerant to urea-induced denaturation^24^. In contrast, the surface hydrophobicity of carp myosin increased upon the addition of urea. The results of this study corroborate observations made previously. Banded houndshark light chains may stabilize myosin, especially the head region responsible for ATPase activity, in the presence of urea by retaining their secondary structure.

Recently, ATP was reported as a candidate for the counteractant against urea-induced denaturation of proteins. ATP suppresses urea-induced denaturation of myosin Ca^2+^-ATPase of scalloped hammerhead shark that contains urea and red sea bream that contains only a low level of urea^31^. ATP is a biological hydrotrope that maintains the solubility of hydrophobic proteins^32,33^. Besides TMAO, ATP may also play a role in counteracting urea-induced denaturation of proteins.

In this study, we showed that the shark myosin light chain has intrinsic urea-resistibility. We are planning to elucidate areas or amino acids responsible for the urea-resistibility using proteins synthesized with the amino acid substitution or deletion system.

## Materials and methods

All experimental protocols were approved by Mie University.

### Materials

We obtained live specimens of banded houndshark *Triakis scyllium* captured off the Shima peninsula courtesy of a private aquarium Shima Marineland (Shima, Mie, Japan). After decapitation, the dorsal fast muscle was carefully excised, transported to our laboratory on ice and used immediately to prepare myosin light chains. Alternatively, the muscle was coarsely minced, mixed with an equal volume of glycerol and stored at − 20 °C until use. Live carp *Cyprinus carpio* were purchased from a fish market near Mie University. We used the freshly dissected carp dorsal fast muscle as a reference in the CD analysis. All methods were performed in accordance with the relevant guidelines and regulations of Mie University.

### Preparation of myosin light chains

All procedures were performed at 4 °C or on ice unless otherwise described. Banded houndshark dorsal fast muscle was homogenized in Buffer A (25 mM potassium phosphate, pH 6.9), followed by centrifugation at 4200 × *g* for 5 min. The same procedure was repeated five times against the obtained pellet to obtain shark myofibrils as a pellet. The myofibril pellet was suspended with three volumes of Buffer B (350 mM potassium phosphate, pH 6.9, 1 mM EDTA, 10 mM sodium pyrophosphate, 10 mM MgCl_2_, 2 mM ATP, 0.01% (v/v) 2-mercaptoethanol) to elute myofibrillar proteins on ice for 15 min. After centrifugation at 8200×*g* for 10 min, the supernatant was filtered with a double-layered gauze. The filtrate was fractionated by 40–48% ammonium sulfate saturation. The fraction was suspended with a small amount of Buffer A and dialyzed against the same buffer overnight. The dialyzed sample was subjected to isoelectric precipitation at pH 6.4, followed by dilution with 10 volumes of cold distilled water. After ultracentrifugation at 20,400 × *g* for 30 min, crude myosin was obtained as a precipitate. The crude myosin was dialyzed against Buffer C (40 mM sodium pyrophosphate, pH 7.5, 1 mM EDTA, 0.01% (v/v) 2-mercaptoethanol). The dialysate was diluted with an equal volume of Buffer D (50 mM Tris–HCl, pH 8.0, 8 M Urea, 0.5 M KCl, 10 mM EDTA, 0.01% (v/v) 2-mercaptoethanol), and the sample stirred for 1 h at room temperature to detach myosin light chains from myosin heavy chains. Myosin heavy chain was precipitated by diluting the solution with 10 volumes of cold distilled water and successive centrifugation at 8200×*g* for 10 min. The supernatant was fractionated by 45–75% ammonium sulfate saturation. After centrifugation at 20,400×*g* for 30 min, a precipitate was obtained as the crude myosin light chain mixture. The preparation of carp myosin light chains followed the same procedure, except 40–50% ammonium sulfate saturation was used instead of 40–48% saturation, as described above. The crude myosin light chain mixture was subjected to DEAE-Toyopearl 650 M (20 × 800 mm) equilibrated with Buffer E (10 mM Tris–HCl, pH 7.5, 4 M Urea, 15 mM KCl, 0.07% (v/v) 2-mercaptoethanol) after dialysis against the same buffer overnight. The adsorbed proteins were eluted by a linear gradient up to 0.15 M KCl. To separate A1 and A2 from DTNB, the fraction containing those proteins was loaded onto a TSK gel phenyl-5PW column (7.5 × 75 mm) equilibrated with Buffer F (0.5 M ammonium sulfate for A1, 0.3 M ammonium sulfate for A2, 0.1 M potassium phosphate, pH 7.0). The adsorbed proteins were eluted using a linear gradient of ammonium sulfate down to 0 M.

### N-terminal protein sequencing of shark myosin A1 and A2 light chains

Protein sequencing was performed by a conventional method to identify the purified proteins as A1-LC and A2-LC^34^. The purified A1-LC and A2-LC were digested with *N*-*p*-tosyl-L-phenylalanine chloromethyl ketone (TPCK) treated trypsin, separated on SDS-PAGE and electroblotted onto a polyvinylidene difluoride (PVDF) membrane. The coomassie brilliant blue stained bands were subjected to protein sequencing using a protein sequencer (model 476A, Applied Biosystems Japan, Tokyo, Japan).

### Circular Dichroism (CD) analysis of shark and carp myosin A1 and A2 light chains

Purified shark A1-LC (0.017 mg/mL) was treated with 0–2.0 M urea in 50 mM potassium phosphate buffer (pH 7.0) containing 0.5 M KCl and 0.1 mM dithiothreitol (DTT) at 0 °C for 24 h. CD spectral profiles of the treated shark A1-LC was measured at room temperature using a CD spectropolarimeter (J-720 M, JASCO, Tokyo, Japan) equipped with a quartz cylindrical cell (20 × 10 mm). Each sample was scanned four times, and the averaged spectra were obtained. Molar ellipticity was calculated using spectral profiles with computer software (J700 system software) provided by the CD manufacturer. The α-helical content was also calculated with the same system. Shark A2-LC, carp A1-LC and carp A2-LC were analyzed using the same approach.

### cDNA cloning of shark myosin A1, A2 and DTNB light chains

We raised antibodies against shark A1-LC, A2-LC and myosin with those purified proteins described above using rabbit or mouse. These antiserums were used for immuno-screening as follows. The cDNA library of the banded houndshark *Triakis scyllium* fast skeletal muscle was constructed with the SuperScript Lambda System for cDNA Synthesis and λ Cloning Kit (Gibco BRL, Gaithersburg, MD, USA) using mRNA purified from the total RNA, according to the manufacturer’s manual. Immuno-screening was carried out with the anti-shark A1-LC antiserum to isolate cDNA clones encoding the *Triakis scyllium* A1-LC gene (*TsMLC-A1*). For the *Triakis scyllium* A2-LC gene (*TsMLC-A2*) and DTNB-LC gene (*TsMLC-DTNB*), we used the anti-shark A2-LC and myosin antiserums, respectively. Positive cDNA clones obtained by respective screenings were sequenced by a conventional method. The nucleotide sequences of *TsMLC-A1*, *TsMLC-A2* and *TsMLC-DTNB* were registered with the DDBJ/EMBL/GenBank databases as LC685050, LC685051 and LC685052, respectively.

### Comparison of shark myosin light chains with those from teleosts and other vertebrates

The deduced amino acid sequences of the banded houndshark myosin A1-LC, A2-LC and DTNB-LC were compared with those from teleosts and other vertebrates using Clustal W^35^. We used the following data: carp *Cyprinus carpio* (D85139, D85140, D85141), skipjack tuna *Katsuwonus pelamis* (AB042037, AB042038, AB042039), bluefin tuna *Thunnus orientalis* (AB042034, AB042035, AB042036), sardine *Sardinops melanostictus* (AB042049, AB042050, AB042051), anchovy *Engraulis japonica* (AB042052, AB072799, AB042053), horse mackerel *Trachurus japonicus* (AB042046, AB042047, AB042048), walleye pollack *Gadus chalcogramma* (AB042054, AB051825, AB051824), flying fish *Cypselurus agoo* (AB042043, AB042044, AB042045), white croaker *Pennahia argentata* (AB042040, AB042041, AB042042), zebrafish *Danio rerio* (AB042028), rabbit *Oryctolagus cuniculus* (X54041, X54044), rat *Rattus norvegicus* (AH003510), mouse *Mus musculus* (NM_021285, NM_001113387), chicken *Gallus gallus* (J00888, M11030) and African clawed frog *Xenopus laevis* (NM_001086783).

### Hydrophilicity analysis of shark and carp myosin A1 light chains

Hydrophilicity values of the shark and carp myosin A1-LC were calculated using ProtScale (https://web.expasy.org/protscale/) and amino acid scale values by Hopp & Woods: Ala: − 0.500; Arg: 3.000; Asn: 0.200; Asp: 3.000; Cys: − 1.000; Gln: 0.200; Glu: 3.000; Gly: 0.000; His: − 0.500; Ile: − 1.800; Leu: − 1.800; Lys: 3.000; Met: − 1.300; Phe: − 2.500; Pro: 0.000; Ser: 0.300; Thr: − 0.400; Trp: − 3.400; Tyr: − 2.300; and Val: − 1.500 ^36^.

## Supplementary Information

Supplementary Figures.

## Data Availability

The nucleotide sequences of *TsMLC-A1*, *TsMLC-A2* and *TsMLC-DTNB* were registered with the DDBJ/EMBL/GenBank databases as LC685050, LC685051 and LC685052, respectively. The authors confirm that the data supporting the findings of this study are available within the article and its supplementary materials.

## References

[1] Holmes WN, Donaldson EM, Hoar WS, Randall DJ (1969). The body compartments and the distribution of electrolytes. Fish Physiology.

[2] Watabe S, Ochiai Y, Kanoh S, Hashimoto K (1983). Proximate and protein compositions of requiem shark muscle. Nippon Suisan Gakkai Shi.

[3] Hermans J (1966). The effect of protein denaturants on the stability of the α Helix1. J. Am. Chem. Soc..

[4] Watlaufer DB, Malik SK, Stoller L, Coffin RL (1964). Nonpolar group participation in the denaturation of proteins by urea and guanidinium salts. Model compound studies. J. Am. Chem. Soc..

[5] Finer EG, Franks F, Tait MJ (1972). Nuclear magnetic resonance studies of aqueous urea solutions. J. Am. Chem. Soc..

[6] Street TO, Bolen DW, Rose GD (2006). A molecular mechanism for osmolyte-induced protein stability. Proc. Natl. Acad. Sci. U. S. A..

[7] Hua L, Zhou R, Thirumalai D, Berne BJ (2008). Urea denaturation by stronger dispersion interactions with proteins than water implies a 2-stage unfolding. Proc. Natl. Acad. Sci. U. S. A..

[8] Lim WK, Rösgen J, Englander SW (2009). Urea, but not guanidinium, destabilizes proteins by forming hydrogen bonds to the peptide group. Proc. Natl. Acad. Sci. U. S. A..

[9] Zigman S, Munro J, Lerman S (1965). Effect of urea on the cold precipitation of protein in the lens of the dogfish. Nature.

[10] Bonaventura J, Bonaventura C, Sullivan B (1974). Urea tolerance as a molecular adaptation of elasmobranch hemoglobins. Science.

[11] Yancey PH, Somero GN (1978). Urea-requiring lactate dehydrogenases of marine elasmobranch fishes. J. Comp. Physiol..

[12] Yancey, P. H. Organic osmotic effectors in cartilaginous fishes. In: *Transport Processes, Iono- and Osmoregulation* 424–436 (Springer Berlin Heidelberg, 1985).

[13] Bennion BJ, Daggett V (2004). Counteraction of urea-induced protein denaturation by trimethylamine N-oxide: a chemical chaperone at atomic resolution. Proc. Natl. Acad. Sci. U. S. A..

[14] Ganguly P, Boserman P, van der Vegt NFA, Shea J-E (2018). Trimethylamine N-oxide counteracts urea denaturation by Inhibiting protein-urea preferential interaction. J. Am. Chem. Soc..

[15] Kanoh S, Kitamura M, Horie Y (2001). Effects of urea and trimethylamine-N-oxide on ATPase of requiem shark myofibril and its constituents. Fish. Sci..

[16] Kanoh S, Niwa E, Osaka Y, Watabe S (1999). Effects of urea on actin-activated Mg^2+^-ATPase of requiem shark myosin. Comp. Biochem. Physiol. B Biochem. Mol. Biol..

[17] Nabeshima Y, Fujii-Kuriyama Y, Muramatsu M, Ogata K (1984). Alternative transcription and two modes of splicing results in two myosin light chains from one gene. Nature.

[18] Robert B, Daubas P, Akimenko MA (1984). A single locus in the mouse encodes both myosin light chains 1 and 3, a second locus corresponds to a related pseudogene. Cell.

[19] Periasamy M, Strehler EE, Garfinkel LI (1984). Fast skeletal muscle myosin light chains 1 and 3 are produced from a single gene by a combined process of differential RNA transcription and splicing. J. Biol. Chem..

[20] Libera LD, Carpene E, Theibert J, Collins JH (1991). Fish myosin alkali light chains originate from two different genes. J. Muscle Res. Cell Motil..

[21] Hirayama Y, Kanoh S, Nakaya M, Watabe S (1997). The two essential light chains of carp fast skeletal myosin, LC1 and LC3, are encoded by distinct genes and change their molar ratio following temperature acclimation. J. Exp. Biol..

[22] Ishizaki S, Masuda Y, Tanaka M, Watabe S (2002). Structure and function of fish fast skeletal muscle myosin light chains. Fish. Sci..

[23] Heissler SM, Sellers JR (2014). Myosin light chains: teaching old dogs new tricks. BioArchitecture.

[24] Kanoh S, Taniguchi J, Yamada T, Niwa E (2000). Effects of urea on surface hydrophobicity of requiem shark myosin. Fish. Sci..

[25] Lowey S, Waller GS, Trybus KM (1993). Skeletal muscle myosin light chains are essential for physiological speeds of shortening. Nature.

[26] Kanoh S, Watabe S, Takewa T, Hashimoto K (1985). Urea-resistibility of myofibrillar proteins from the requiem shark Triakis scyllia. Bull. Jpn. Soc. Sci. Fish..

[27] Weber RE (1983). TMAO (trimethylamine oxide)-independence of oxygen affinity and its urea and ATP sensitivities in an elasmobranch hemoglobin. J. Exp. Zool..

[28] Burg MB, Peters EM (1997). Urea and methylamines have similar effects on aldose reductase activity. Am. J. Physiol..

[29] Grundy JE, Storey KB (1994). Urea and salt effects on enzymes from estivating and non-estivating amphibians. Mol. Cell Biochem..

[30] Mashino T, Fridovich I (1987). Effects of urea and trimethylamine-N-oxide on enzyme activity and stability. Arch. Biochem. Biophys..

[31] Ogata Y, Kimura I (2019). Adenosine triphosphate suppresses urea-induced denaturation of shark myosin calcium adenosine triphosphatase. Fish. Sci..

[32] Patel A, Malinovska L, Saha S (2017). ATP as a biological hydrotrope. Science.

[33] Rice AM, Rosen MK (2017). ATP controls the crowd. Science.

[34] Matsudaira P (1987). Sequence from picomole quantities of proteins electroblotted onto polyvinylidene difluoride membranes. J. Biol. Chem..

[35] Larkin MA, Blackshields G, Brown NP (2007). Clustal W and Clustal X version 2.0. Bioinformatics.

[36] Hopp TP, Woods KR (1981). Prediction of protein antigenic determinants from amino acid sequences. Proc. Natl. Acad. Sci. USA.

