# Supplementary material for: Myosin light chain of shark fast skeletal muscle exhibits intrinsic urea-resistibility
Source: Sci Rep. 2023 Mar 25;13:4909. doi: 10.1038/s41598-023-32228-w (PMC10039937; doi:10.1038/s41598-023-32228-w)
Supplement: Supplementary file 1 — Supplementary Figures. [file 41598_2023_32228_MOESM1_ESM.pdf]

Supplementary Materials

Myosin light chain of shark fast skeletal muscle exhibits intrinsic urea-resistibility

Satoshi Kanoh[1], Takayuki Noma[1,2], Hirotaka Ito[1,3], Masatomo Tsureyama[1,4], Daisuke Funabara[1]*

[1]Graduate School of Bioresources, Mie University, Tsu, Mie 514-8507, Japan
[2]Current address: KOGAKKAN HIGH SCHOOL, Ise, Mie 516−8577, Japan
[3]Current address: ASGEN Pharmaceutical Co., Ltd., Mizunami, Gifu, 509-6104, Japan
[4]Current address: Kracie Foods, Ltd., Minato, Tokyo, 108-8080, Japan

Satoshi Kanoh: kanoh@bio.mie-u.ac.jp
Takayuki Noma: takayuki@kogakkan-u.ac.jp
Hirotaka Ito: h_itou@asgen.co.jp
Masatomo Tsureyama: m.tsureyama@kracie.co.jp
Daisuke Funabara: funabara@bio.mie-u.ac.jp

*Corresponding author: Daisuke Funabara

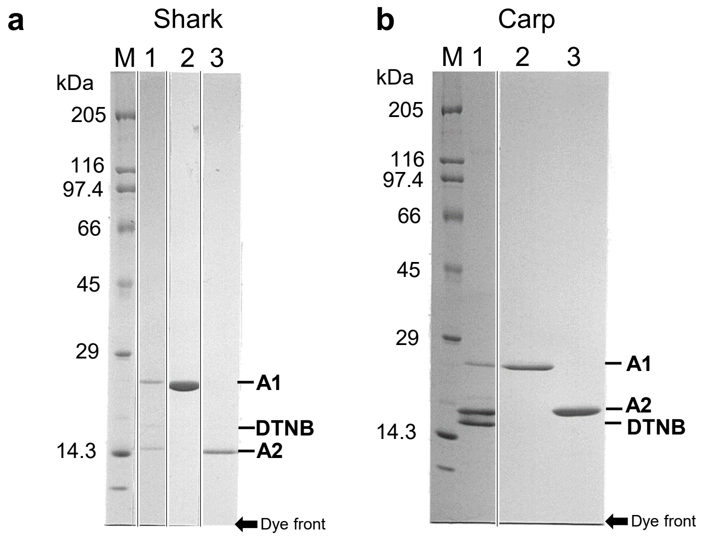

Figure S1 SDS-PAGE patterns of the purified myosin light chains. a, banded houndshark *Triakis scyllium* myosin light chains. b, carp *Cyprinus capio* myosin light chains. M, molecular markers; lane 1, total myosin light chains; lane 2, myosin A1-LC; lane 3, myosin A2-LC. All lanes of each photo originated from the same SDS-gel photo.

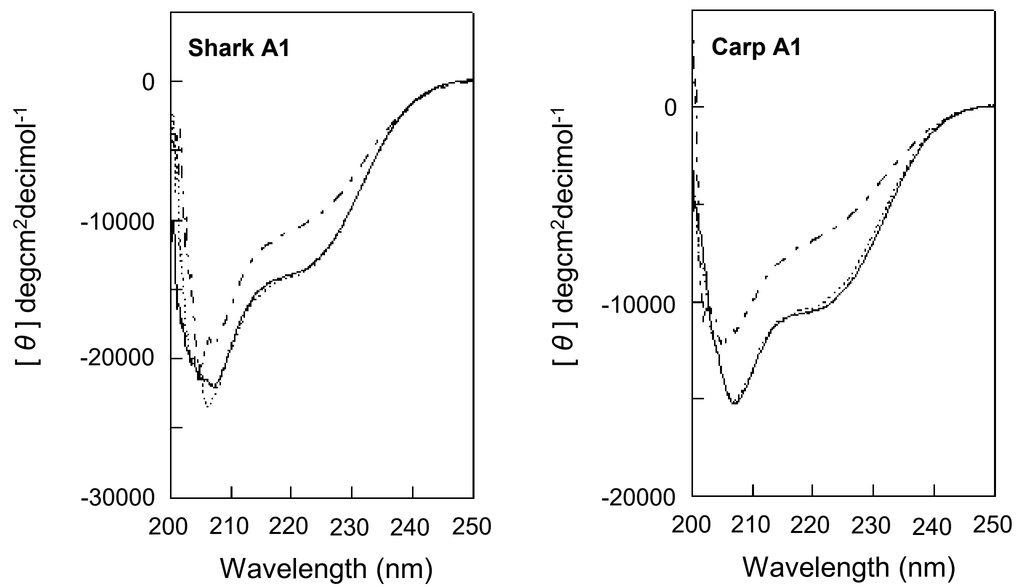

Figure S2 CD spectra of shark (left) and carp (right) myosin A1-LCs in the absence of urea. Measurement was performed in 50mM Tris-HCl (pH8.0) containing 0.6 M KCl,5 mM $MgCl_2$ and 1mM DTT. Solid lines indicate the CD spectra at 26 ℃, dash-dotted lines at 60 ℃, and dotted lines at 26 ℃ after the measurement at 60 ℃. Note that the measurements were conducted in different conditions from the CD analysis in this paper.

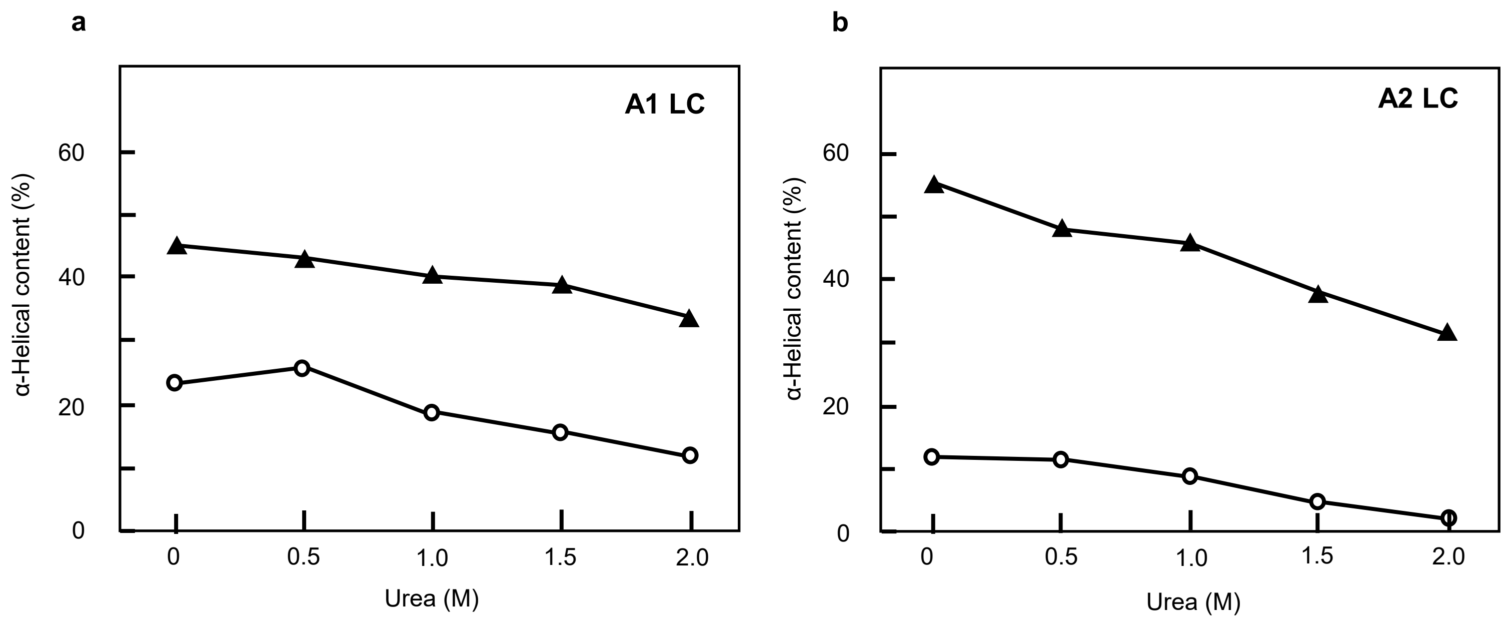

Figure S3 Changes in the α-helical content of shark and carp myosin A1- and A2-LCs in the presence of urea. (a) myosin A1-LC. (b) myosin A2-LC. Open circles and filled triangles represent the data from shark and carp, respectively. The relative α-helical content shown in Fig. 1 was derived from these data.

```
CACACCAGCAGACTCTAACTAACTCCAGGCGTCTACCTCAACCCATCCCCCAAATGGCAC   60
                                                     M  A        2
CCAAGAAGGATGTAAAGAAGCCAGAACCCAAGGCTGCCGCGGCTCCTGCTCCTGCTGCTG  120
P  K  K  D  V  K  K  P  E  P  K  A  A  A  A  P  A  P  A  A      22
CTCCCCCACCACCCGAACCACCCAAACCCAAGGAGCCATCTGTCGACTTGTCCAAGGTCA  180
A  P  P  P  P  E  P  P  K  P  K  E  P  S  V  D  L  S  K  V      42
AGATCGAGTTCAGCGCCGAGCAGCAGGAAGACTTCAAGGAGGCTTTCCTCCTCTTTGACA  240
K  I  E  F  S  A  E  Q  Q  E  D  F  K  E  A  F  L  L  F  D      62
GAATTGGTAATTCCAAGATTACCTATGGCCAGGTTGCGGATGTCATGCGCGCTCTCGGCC  300
R  I  G  N  S  K  I  T  Y  G  Q  V  A  D  V  M  R  A  L  G      82
AGAATCCAACCAATGCTGAGGTGAAGAAAATCCTGAACAACCCCAGCACTGAGGATATGA  360
Q  N  P  T  N  A  E  V  K  K  I  L  N  N  P  S  T  E  D  M     102
CCAGCAAAGCCATTGAATTTGACCAGTTCCTGCCCATGCTCCAAACCATGGCCAACAACA  420
T  S  K  A  I  E  F  D  Q  F  L  P  M  L  Q  T  M  A  N  N     122
AGGAGCAGGGCTCATATGAAGACTTTGTTGAGGGTCTGCGTGTCTTTGACAAGGAAGGCA  480
K  E  Q  G  S  Y  E  D  F  V  E  G  L  R  V  F  D  K  E  G     142
ACGGCACTGTGATGGGGGCTGAGCTCCGCCACGTTCTGGCTACACTGGGTGAGAAGTTGA  540
N  G  T  V  M  G  A  E  L  R  H  V  L  A  T  L  G  E  K  L     162
CAGAGGATCAGGTAGAACAACTGCTGACAGGTCAGGAAGACGCCAATGGCTGCATCAACT  600
T  E  D  Q  V  E  Q  L  L  T  G  Q  E  D  A  N  G  C  I  N     182
ATGAGGCTTTTGTCAAACACATCATGTCTGTTTAAAATGGATCCCCATGAATTGGTGCAT  660
Y  E  A  F  V  K  H  I  M  S  V  *                             193
ATGGTTCTAAATGGCTGAACACCTGGCAGGGCCAAAACATCAGACCCTGGAACTTGTGGA  720
TTTCTGACCAATGCAAACAGACTCCTTTTAAAGCCTGTACCAGAACCGCTTTGTTTTTTT  780
TTACTTGTATCCAATGGACAGTATTTCAAAACAACAAAATGAGGAAGAACAGGAAAAACC  840
TTGACGTTTGCGGTGGCAAACCTGATGGACAAAACTGTTTGGTTTGTCCATGATGTTTGG  900
TGTGGTTGGAAAGTGCACGTGGCAGTCAACATCCAATAAAGTCTCAGTGGTCAATCTGTG  960
GCTGTATCATCCATATTACATCTCCACATGCAGCAGTCTTCACATGTTAACTCTGTACAG 1020
TGTACTTCAAGAAAATAACACAAAATGTCAACAAAAAAAAAAAAAAAAAA           1070
```

Figure S4 Nucleotide and deduced amino acid sequences of banded houndshark *Triakis scyllium* myosin A1-LC. Underlined amino acid sequences are derived from internal N-terminal amino acid sequencing of myosin A1-LC purified from banded houndshark fast skeletal muscle. The numbering on the right side of the sequences denotes the nucleotide sequence from the 5'-end and amino acid sequence from the N-terminus. The asterisk denotes the stop codon

```
TGCTATTCCTCTGCCGATCCGCAGTCATGTCATTCTCAGCAGACGAGGTGGCCGACTTCA   60
                          M  S  F  S  A  D  E  V  A  D  F      11
AGGAGGCTTTCCTCCTCTTTGACAGAATTGGTAATTCCAAGATTACCTATGGCCAGGTTG  120
K  E  A  F  L  L  F  D  R  I  G  N  S  K  I  T  Y  G  Q  V     31
CGGATGTCATGCGCGCTCTCGGCCAGAATCCAACCAATGCTGAGGTGAAGAAAATCCTGA  180
A  D  V  M  R  A  L  G  Q  N  P  T  N  A  E  V  K  K  I  L     51
ACAACCCCAGCACTGAGGATATGACCAGCAAAGCCATTGAATTTGACCAGTTCCTGCCCA  240
N  N  P  S  T  E  D  M  T  S  K  A  I  E  F  D  Q  F  L  P     71
TGCTCCAAACCATGGCCAACAACAAGGAGCAGGGCTCATATGAAGACTTTGTTGAGGGTC  300
M  L  Q  T  M  A  N  N  K  E  Q  G  S  Y  E  D  F  V  E  G     91
TGCGTGTCTTTGACAAGGAAGGCAACGGCACTGTGATGGGGGCTGAGCTCCGCCACGTTC  360
L  R  V  F  D  K  E  G  N  G  T  V  M  G  A  E  L  R  H  V    111
TGGCTACACTGGGTGAGAAGTTGACAGAGGATCAGGTAGAACAACTGCTGACAGGTCAGG  420
L  A  T  L  G  E  K  L  T  E  D  Q  V  E  Q  L  L  T  G  Q    131
AAGACGCCAATGGCTGCATCAACTATGAGGCTTTTGTCAAACACATCATGTCTGTTTAAA  480
E  D  A  N  G  C  I  N  Y  E  A  F  V  K  H  I  M  S  V  *    150
ATGGATCCCCATGAATTGGTGCATATGGTTCTAAATGGCTGAACACCTGGCAGGGCCAAA  540
ACATCAGACCCTGGAACTTGTGGATTTCTGACCAATGCAAACAGACTCCTTTTAAAGCCT  600
GTACCAGAACCGCTTTGTTTTTTTTTACTTGTATCCAATGGACAGTATTTCAAAACAACA  660
AAATGAGGAAGAACAGGAAAAACCTTGACGTTTGCGGTGGCAAACCTGATGGACAAAACT  720
GTTTGGTTTGTCCATGATGTTTGGTGTGGTTGGAAAGTGCACGTGGCAGTCAACATCCAA  780
TAAAGTCTCAGTGGTCAATCTGTGGCTGTATCATCCATATTACATCTCCACATGCAGCAG  840
TCTTCACATGTTAACTCTGTACAGTGTACTTCAAGAAAATAACACAAAATGTCAACAAAA  900
AAAAAAAAAAAAAAAAAAA                                           919
```

Figure S5 Nucleotide and deduced amino acid sequences of banded houndshark *Triakis scyllium* myosin A2-LC. The underlined sequence indicates the amino acid sequence derived from internal N-terminal amino acid sequencing of myosin A2-LC purified from banded houndshark fast skeletal muscle. The numbering on the right side denotes the nucleotide sequence from the 5'-end and amino acid sequence from the N-terminus. The asterisk denotes the stop codon

```
AGAGAGGCCAGGGATTAGACATGTCACCCAAGCGAGCAAAGAAGAGGGCAGCTGAGGGCT    60
                    M  S  P  K  R  A  K  K  R  A  A  E  G       13
CCTCCAATGTCTTCTCCATGTTCGACCAGACCCAGATCCAGGAGTTCAAGGAGGCATTCA   120
S  S  N  V  F  S  M  F  D  Q  T  Q  I  Q  E  F  K  E  A  F      33
CTGTTATTGACCAGAACAGAGATGGTATCATCGGTAAGGATGACCTTGCTGGCACATTCG   180
T  V  I  D  Q  N  R  D  G  I  I  G  K  D  D  L  A  G  T  F      53
CTGCAGTGGGCCGAATGAATGTTAAGGGTGATGAGTTGGAAGCCATGATCAAAGAAGCTC   240
A  A  V  G  R  M  N  V  K  G  D  E  L  E  A  M  I  K  E  A      73
CCGGCCCCATCAATTTCACTGTCTTCCTCACCATGTTCGGCGAGAAACTCAAAGGCGCTG   300
P  G  P  I  N  F  T  V  F  L  T  M  F  G  E  K  L  K  G  A      93
ACCCCGAGGACGTGATCATGGCAGCTTTCAAAATTCTCGACCATGAGGGCAAAGGCAGCT   360
D  P  E  D  V  I  M  A  A  F  K  I  L  D  H  E  G  K  G  S     113
TAAAGAAAGCTTTCCTTGAAGAAATGTTGACTACCCAGTGCGACAGGTTCACCCCAGAGG   420
L  K  K  A  F  L  E  E  M  L  T  T  Q  C  D  R  F  T  P  E     133
AGATGAAGAATCTCTGGGCAGCCTTCCCTCCAGATGTTGCTGGCAACGTGGACTACAAGA   480
E  M  K  N  L  W  A  A  F  P  P  D  V  A  G  N  V  D  Y  K     153
ACATCTGCTACGTCATCACACACGGTGAGGAGAAAGAAGGTGAATAAGGAGCCCACGGGG   540
N  I  C  Y  V  I  T  H  G  E  E  K  E  G  E  *                168
AGAGCCAAGATCAAATCTCCATGACCACAGAACTCCGCATCATCCCCCCTCCTGCCCCCA   600
CTATTGCCCTGGATTGGAACACCCCTCTGGCAACCGGGGGCAGGCGGGGAGAGAGAGAGG   660
GAGG                                                           664
```

Figure S6 Nucleotide and deduced amino acid sequences of banded houndshark *Triakis scyllium* myosin DTNB-LC. The numbers on the right side represent the nucleotide sequence from the 5'-end and amino acid residues from the N-terminus. The asterisk denotes the stop codon

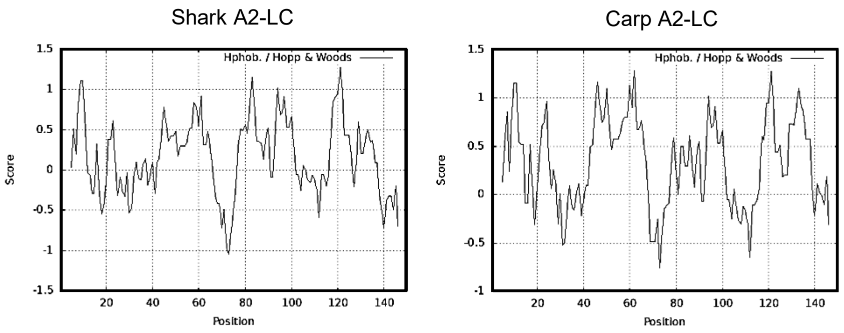

Figure S7 Hydrophicility analysis of the A2-LCs of banded houndshark and carp. Hydrophilicity values of the shark (left) and carp (right) myosin A2-LC were calculated using ProtScale (https://web.expasy.org/protscale/) and amino acid scale values by Hopp & Woods: Ala: –0.500; Arg: 3.000; Asn: 0.200; Asp: 3.000; Cys: –1.000; Gln: 0.200; Glu: 3.000; Gly: 0.000; His: –0.500; Ile: –1.800; Leu: –1.800; Lys: 3.000; Met: –1.300; Phe: –2.500; Pro: 0.000; Ser: 0.300; Thr: –0.400; Trp: –3.400; Tyr: –2.300; and Val: –1.500
